# Resistance of parvalbumin to gastrointestinal digestion is required for profound and long‐lasting prophylactic oral tolerance

**DOI:** 10.1111/all.13994

**Published:** 2019-10-03

**Authors:** Raphaela Freidl, Antonia Gstöttner, Ulrike Baranyi, Ines Swoboda, Frank Stolz, Margarete Focke‐Tejkl, Thomas Wekerle, Ronald van Ree, Rudolf Valenta, Birgit Linhart

**Affiliations:** ^1^ Division of Immunopathology, Department of Pathophysiology and Allergy Research, Center for Pathophysiology, Infectiology and Immunology Medical University of Vienna Vienna Austria; ^2^ Cardiac Surgery Research Laboratory, Department of Surgery Medical University of Vienna Vienna Austria; ^3^ Biomay AG Vienna Austria; ^4^ Section of Transplantation Immunology, Department of Surgery Medical University of Vienna Vienna Austria; ^5^ Departments of Experimental Immunology and of Otorhinolaryngology Academic Medical Center Amsterdam Netherlands; ^6^ NRC Institute of Immunology FMBA of Russia Moscow Russia; ^7^ Laboratory for Immunopathology, Department of Clinical Immunology and Allergy Sechenov First Moscow State Medical University Moscow Russia; ^8^ Present address: Molecular Biotechnology Section, University of Applied Sciences, Campus Vienna Biocenter Vienna Austria

**Keywords:** allergen, allergy, food allergy, oral tolerance induction, parvalbumin

## Abstract

**Background:**

Early introduction of food allergens into children's diet is considered as a strategy for the prevention of food allergy. The major fish allergen parvalbumin exhibits high stability against gastrointestinal digestion. We investigated whether resistance of carp parvalbumin to digestion affects oral tolerance induction.

**Methods:**

Natural Cyp c 1, nCyp c 1, and a gastrointestinal digestion‐sensitive recombinant Cyp c 1 mutant, mCyp c 1, were analyzed for their ability to induce oral tolerance in a murine model. Both antigens were compared by gel filtration, circular dichroism measurement, *in vitro* digestion, and splenocyte proliferation assays using synthetic Cyp c 1‐derived peptides. BALB/c mice were fed once with high doses of nCyp c 1 or mCyp c 1, before sensitization to nCyp c 1. Immunological tolerance was studied by measuring Cyp c 1‐specific antibodies and cellular responses by ELISA, basophil activation, splenocyte proliferations, and intragastric allergen challenge.

**Results:**

Wild‐type and mCyp c 1 showed the same physicochemical properties and shared the same major T‐cell epitope. However, mCyp c 1 was more sensitive to enzymatic digestion *in vitro* than nCyp c 1. A single high‐dose oral administration of nCyp c 1 but not of mCyp c 1 induced long‐term oral tolerance, characterized by lack of parvalbumin‐specific antibody and cellular responses. Moreover, mCyp c 1‐fed mice, but not nCyp c 1‐fed mice developed allergic symptoms upon challenge with nCyp c 1.

**Conclusion:**

Sensitivity to digestion in the gastrointestinal tract influences the capacity of an allergen to induce prophylactic oral tolerance.

## Graphical abstract

The calcium‐binding protein parvalbumin, a major and cross‐reactive allergen for fish allergic patients, induces robust and long‐lasting immunological and clinical oral tolerance in a murine model of fish allergy. A recombinant parvalbumin mutant, that resembled the wild‐type parvalbumin regarding biochemical and immunological properties, but was more sensitive to *in vitro* digestion, failed to induce oral tolerance. Sensitivity to digestion in the gastrointestinal tract influences the capacity of an allergen to induce prophylactic oral tolerance.

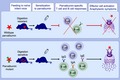

**Abbreviations**
AIT: allergen‐specific immunotherapy
Cpm: counts per minute
i.g.: intragastric
kDa: kilo Dalton
mCyp c 1: recombinant mutant Cyp c 1
MW: molecular weight
nCyp c 1: natural Cyp c 1
OD: optical density
OIT: oral immunotherapy
RBL: rat basophil leukemia
rCyp c 1: recombinant wild‐type Cyp c 1.01
s.c.: subcutaneous
SIT: allergen‐specific immunotherapy
T: temperature
β‐ME: beta‐mercaptoethanol

## 1 INTRODUCTION

Food allergy represents one of the important clinical manifestations of IgE‐associated allergy. It often starts in early childhood and can induce severe and life‐threatening anaphylaxis. Potent allergen sources are peanuts, tree nuts, cow's milk, egg, soy, wheat, shellfish, and fish.^1^, ^2^ Diagnosis of the disease‐causing food allergens is extremely important because it guides allergen‐specific forms of treatment, such as avoidance, diet, introduction of hypoallergenic formulas, and allergen‐specific immunotherapy often performed by the oral route (ie, oral allergen‐specific immunotherapy, OIT).^3^, ^4^, ^5^ In addition, several clinical studies indicate that early introduction of allergen‐containing food into the diet of sensitized but not yet allergic children may prevent the development of food allergy.^6^, ^7^ The development of early allergen‐specific forms for the prevention of allergy such as oral tolerance induction and/or early allergen‐specific immunotherapy (AIT) has become an important topic because it may prevent allergic sensitization, the transition from silent sensitization to symptomatic allergy and the progression from mild to severe forms of allergy especially early in childhood.^8^, ^9^, ^10^, ^11^ Fish represents one of the most important food allergen sources which can induce severe anaphylactic reactions.^12^ The calcium‐binding protein parvalbumin has been identified as the major and cross‐reactive allergen in different fish species and is available as recombinant allergen to identify individuals with specific IgE sensitization.^13^ We have developed a recombinant mutant of carp parvalbumin, mCyp c 1, which differs from the wild‐type allergen only in four amino acids but shows strongly reduced allergenic activity.^14^, ^15^ mCyp c 1 has been used for subcutaneous AIT (SCIT) and induced allergen‐specific blocking antibodies (Clinicaltrials.gov identifier: NCT02017626 and NCT02382718).^16^, ^17^, ^18^ Using a mouse model for fish allergy, we have recently shown that the passive administration of mCyp c 1‐specific IgG antibodies reduced symptoms of fish allergy^19^ similar as has been shown in a clinical trial for Fel d 1‐specific IgG antibodies in cat allergic patients.^20^ Passive immunization with Bet v 1, Phl p 1‐, and Phl p 5‐specific IgG antibodies prevented the development of pollen allergy but the duration of the effect has not been investigated.^21^

In this study, we used wild‐type Cyp c 1 and mCyp c 1 to investigate if early oral administration of the antigens can induce robust and long‐lasting immunological and clinical tolerance in the murine model of fish allergy. In particular, we were interested to study if sensitivity to digestion of the tolerogens may affect the outcome of tolerance induction.

## 2 MATERIALS AND METHODS

### 2.1 Natural and recombinant antigens, synthetic peptides

**Figure 1:**
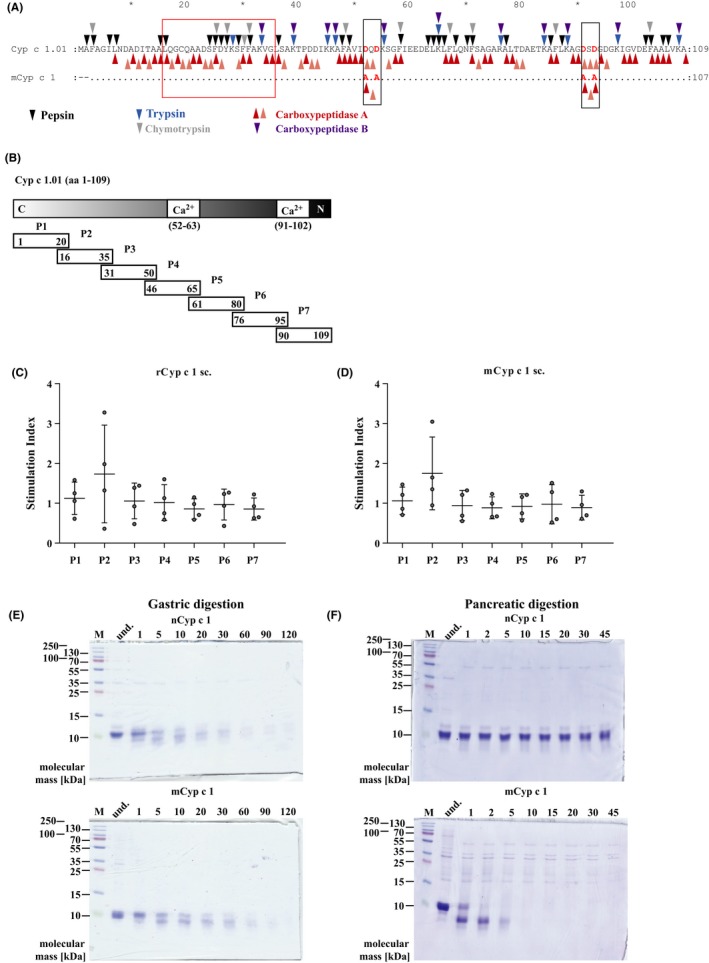
Comparison of mutant Cyp c 1 (mCyp c 1) and wild‐type Cyp c 1. A, Sequence alignment of Cyp c 1.01 and mCyp c 1. Identical amino acids are indicated by dots. Black boxes and red letters indicate the four‐point mutations in mCyp c 1. The major T‐cell‐reactive peptide 2 is boxed in red. Gastric and pancreatic enzyme cleavage sites are depicted in the amino acid sequence of Cyp c 1.01 (upper panel). B, Position of calcium‐binding sites and Cyp c 1‐derived synthetic peptides P1‐P7 within the Cyp c 1 amino acid sequence. C, D, Splenocyte proliferations (y‐axes: stimulation indices) in response to synthetic peptides (x‐axes: P1‐P7; Table S1) after two subcutaneous injections with rCyp c 1 (C) or mCyp c 1 (D) in BALB/c mice. Coomassie‐stained SDS‐gels showing E, the gastric and F, the pancreatic digestion of nCyp c 1 (upper panels) and of mCyp c 1 (lower panels). Samples were taken before digestion (lane: und.) and at different time points during digestion (lanes: 1‐120 and 1‐45 min). Molecular masses (kDa) and molecular mass marker (lane: M) are displayed on the left side

Carp extract was prepared from homogenized carp muscle tissue by extraction in phosphate‐buffered saline (pH 7.4) at 4°C.^22^ For enrichment of natural Cyp c 1 (nCyp c 1), the raw extract was boiled for 30 minutes^22^ and precipitated proteins were removed by filtration. The presence of the Cyp c 1.01 isoform in nCyp c 1 was confirmed by electrospray ionization‐liquid chromatography (mass spectrometry LC‐ESI‐MS/MS).^23^ The recombinant mutant Cyp c 1 (mCyp c 1), based on the Cyp c 1.01 sequence, was expressed in E coli BL21 and purified by ion exchange and hydrophobic interaction chromatography as previously described.^14^ The amino acid sequence of recombinant mutant Cyp c 1 differs from wild‐type Cyp c 1 by 4‐point mutations (*D → A*) in the calcium‐binding sites of the protein (Figure 1A).^14^ Recombinant wild‐type Cyp c 1.01 (rCyp c 1) was obtained from Biomay AG. rCyp c 1 was expressed in E coli and purified by conventional biochemical methods.^13^ Endotoxin levels for nCyp c 1 (<1.35 EU/µg), mCyp c 1 (>100 EU/µg), and rCyp c 1 (0.155 EU/µg) were measured on an Endosafe‐PTS detection system (Charles River Laboratories Int.). An E coli‐expressed, recombinant hypoallergenic hybrid molecule derived from the major timothy grass pollen allergens Phl p 2 and Phl p 6 (hP62) served as control antigen.^24^ Synthetic peptides spanning the Cyp c 1.01 sequence (Table S1) were produced by solid phase peptide chemistry, purified to homogeneity, and characterized by mass spectrometry as described.^19^

### 2.2 *In vitro* digestion assays

Enzyme cleavage sites in nCyp c 1 and mCyp c 1 amino acid sequence were analyzed using ExPASy—PeptideCutter program. Positions for cleavage sites for pepsin (pH > 2, n = 28, black), trypsin (n = 12, blue), chymotrypsin high specificity (n = 11, gray) are indicated in Figure 1A. Further, potential carboxypeptidase A cleavage sites at aromatic and hydrophobic side chains are indicated in red (nCyp c 1: n = 44; mCyp c 1: n = 24) and for other amino acids in light red (nCyp c 1: n = 47; mCyp c 1: n = 21).^25^ Similarly carboxypeptidase B cleavage sites (n = 11) are indicated in the amino acid sequence in purple.^26^

Gastric and pancreatic digestion was mimicked *in vitro* as previously described.^27^, ^28^, ^29^ Briefly, 600 µg of nCyp c 1 and mCyp c 1 were incubated with gastric (pepsin) (Enzynorm; Casella‐Med) or pancreatic (trypsin, chymotrypsin, carboxypeptidase A and B) enzyme solution (Solvay Pharma) at 37°C/300 rpm to mimic peristalsis in the digestive tract. Samples were taken at different time points, aliquots of 5 µg protein were loaded on SDS‐gels for analysis of degradation and gels were stained with Coomassie Brilliant Blue.

### 2.3 Subcutaneous immunization of BALB/c mice using rCyp c 1 or mCyp c 1, splenocyte proliferations

All mouse experiments were approved by the ethical review board of the Medical University of Vienna. Mice were maintained at the Department of Pathophysiology and Allergy Research, Medical University of Vienna, according to the local guidelines for animal welfare. Female BALB/c mice were purchased from Charles River at 6‐8 weeks of age. For studying T‐cell responses to wild‐type and mutant Cyp c 1, groups of mice (n = 4) received two subcutaneous immunizations with 50 µg rCyp c 1 or mCyp c 1 adsorbed to 75 µL aluminum hydroxide (Alu‐Gel‐S; SERVA Electrophoresis GmbH) in a three‐week interval (days 1 and 21).^30^ Mice were sacrificed on day 96 and allergen‐specific splenocyte proliferation was analyzed. For this purpose, single‐cell suspensions were prepared from spleens using a 70‐µm cell strainer sieve (Falcon^TM^; BD Biosciences) under sterile conditions. Cells were seeded into 96‐well round bottom cell culture plates (2 × 10^5^ cells/well) (Corning, Costar) in the presence or absence of stimuli in RPMI medium (Biochrom, Merck), 10% FCS, 0.1 mg/mL Gentamycin, 2 mmol/L L‐Glutamin, 50 µmol/L β‐ME (Gibco^TM^, Thermo Fisher Scientific). Splenocytes were stimulated with 10 µg/mL rCyp c 1 or nCyp c 1 or 1.8 µg/mL of each of the Cyp c 1‐derived peptides or, for control purposes, with 2.5 µg/mL concanavalin A (Sigma‐Aldrich) in triplicates. Cells were grown for 3 days, followed by addition of 0.5 µCi/well ^3^H‐Thymidine (Perkin Elmer). Thymidine incorporation was measured in a β‐radiation counter (MicroBeta TriLux scintillation counter, PerkinElmer). The ratio of the mean counts per minute (cpm) values after antigen stimulation and medium values were calculated as stimulation index (SI) for each mouse.^19^

### 2.4 Prophylactic oral tolerance induction in a mouse model of fish allergy

For tolerance induction experiments, female BALB/c mice were purchased from Charles River at three weeks of age. Groups of mice (n = 8) were fed intragastrically (i.g.) with 10 mg nCyp c 1, or mCyp c 1, resolved in PBS pH 7.4, or PBS on day 1 using a 13 gauge stainless steel feeding needle (Harvard Apparatus). Then mice were immunized twice subcutaneously with 20 µg nCyp c 1 and 20 µg control antigen (hP62) adsorbed to 75 µL aluminum hydroxide (days 5 and 19). The two proteins were administered at two different injection sites in the neck. Control groups either received intragastric gavage or subcutaneous (s.c.) sensitization only. Allergen challenge was performed with 10 mg nCyp c 1 in each mouse group (day 177) and allergic symptoms were recorded. A previously established symptom scoring model for food anaphylaxis was applied.^31^ Body temperature was measured using a digital rectal thermometer shortly before the challenge and for one hour in intervals of 10 minutes (DT‐610B; ATP Instrumentation LTD). The maximal drop of body temperature during the 1‐hour period was used to calculate the maximal drop of body temperature for each mouse. On day 204, mice were sacrificed and splenocyte proliferation assays were performed as described.^19^

### 2.5 Analysis of Cyp c 1‐specific antibody responses

ELISA plates (Nunc Maxisorp) were coated with 3 µg/mL rCyp c 1 in bicarbonate buffer (pH 9.6). Mouse sera dilutions (1:20 IgE; 1:500 IgG1; 1:50 IgG2a, IgG3, IgM, and IgA) were added to the plates and incubated overnight at 4°C. Plates were washed 5 times with PBST and incubated with either rat anti‐mouse IgE, IgG1, IgG2a, IgG3, IgM, or IgA antibody (1:1000; GE Healthcare) overnight at 4°C. Bound antibodies were detected with a HRP‐labeled goat anti‐rat IgG antibody (1:2000; BioLegend). OD values were measured in duplicates and are presented as mean ± SD per mouse group.

### 2.6 Rat basophil leukemia assay

Rat basophil leukemia (RBL)‐2H3 cells were seeded (6 × 10^4^ cells/well) to 96‐well cell culture plates (Corning) and allowed to grow at 37°C (5% CO_2_) for 16 hours. Cells were exposed to serum from each individual mouse (1:10) in triplicates, washed twice with Tyrode's buffer/0.1% BSA and incubated with 0.3 µg/mL rCyp c 1 or control antigen for 1 hour at 37°C (5% CO_2_). Allergic mediator release in cell culture supernatants was detected by the addition of 4‐methylumbelliferyl β‐D‐galactopyranoside (4‐MUG; Sigma‐Aldrich). Cells, which were lysed with 10% v/v Triton X‐100 (Merck Millipore) served as 100% release value. Fluorescence measurement (360‐465 nm) of beta‐hexosaminidase release was performed on an Infinite 200 PRO microplate reader (Tecan). Based on the numeric values measured for the lysed cells, the percentage of beta‐hexosaminidase release from cells loaded with the individual mouse sera was calculated. Percentages of beta‐hexosaminidase release are displayed for each mouse group (mean ± SD).

### 2.7 Statistical analysis

Data were analyzed using GraphPad Prism software 5.0 (GraphPad Software). Significant differences between 2 groups were calculated using a Mann‐Whitney U test. Significant differences between more than 2 groups were calculated using a Kruskal‐Wallis test and Dunn's posttest. Paired data were analyzed by Wilcoxon signed‐rank test. Scatter plots represent mean ± SD. (*) *P*‐value < 0.05, (**) *P*‐value < 0.01, (***) *P*‐value < 0.001.

## 3 RESULTS

### 3.1 nCyp c 1 and mCyp c 1 show similar physicochemical properties and share the major T‐cell epitope

The major fish allergen Cyp c 1 differs from its recombinant mutant, mCyp c 1, only regarding 4 amino acid exchanges (ie, changes of two aspartic acids by alanines) in two calcium‐binding sites (Figure 1A).^14^ The E coli‐expressed recombinant mCyp c 1 and natural Cyp c 1 were purified and subjected to physicochemical characterization. Both molecules migrated as 11 kDa proteins in SDS‐PAGE and were recognized by mCyp c 1‐specific rabbit IgG antibodies (Figure S1A‐C). Gel filtration experiments revealed that both proteins occur as monomers and low molecular weight aggregates (nCyp c 1 peak fractions: 14, 34 kDa; mCyp c 1 peak fractions: 11, 21, 45 kDa). In addition, mCyp c 1 contained low amounts of aggregates in the range of 104 kDa (Figure S1D). Circular dichroism measurements indicated that both proteins were folded, dominated by α‐helices (nCyp c 1: minima at 207 and 220 nm; mCyp c 1: minima at 207 and 221 nm) and were able to refold to their initial shape after heating up to 90°C and cooling down to 20°C (Figure S1E‐F). The mapping of the T‐cell epitopes with overlapping Cyp c 1‐derived peptides in BALB/c mice sensitized with nCyp c 1 and mCyp c 1 indicated that peptide 2 (Figure 1B, Table S1) contained the major T‐cell epitope for nCyp c 1 and mCyp c 1‐sensitized mice. However, the presence of other T‐cell epitopes cannot be completely excluded due to the short overlap of peptides (Figure 1B‐D).

**Figure 2:**
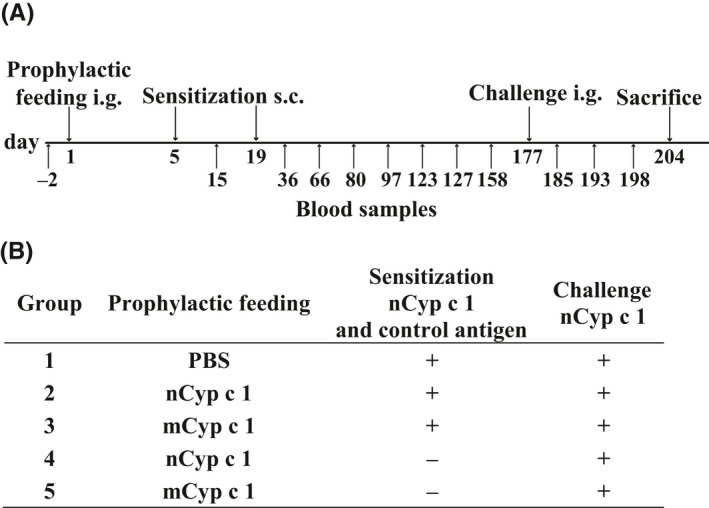
Time course and mouse groups of the prophylactic oral tolerance induction experiments. A, Intragastric (i.g.) gavage of BALB/c mice (day 1) with nCyp c 1, mCyp c 1 or PBS was followed by 2 simultaneous subcutaneous (s.c.) sensitizations with 20 µg nCyp c 1 and 20 µg of an unrelated control antigen adsorbed to aluminum hydroxide or without sensitization (days 5, 19). Allergen challenge (i.g.) with nCyp c 1 was performed on day 177. Blood samples were taken at the indicated days. B, Overview of the 5 mouse groups (n = 8) and the corresponding treatments (prophylactic feeding, sensitization, and challenge)

**Figure 3:**
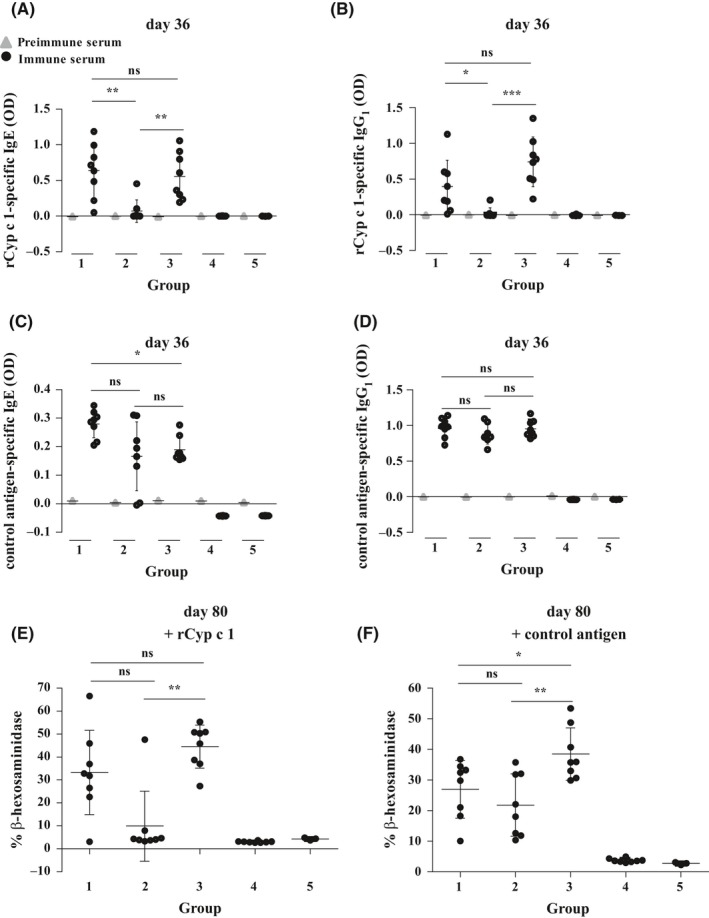
Suppression of Cyp c 1‐specific antibody responses and basophil activation by oral tolerance induction. Comparison of A, rCyp c 1‐specific IgE and B, IgG_1_ and C, control antigen‐specific IgE and D, IgG_1_ levels (y‐axes: OD levels, means ± SD) before sensitization (left, gray triangles) and after sensitization (right, black circles) on day 36 in the mouse groups (x‐axes: 1‐5). E,F, ß‐hexosaminidase release (y‐axis: percentages of total release, means ± SD) from RBL cells loaded with sera from sensitized mice (day 80) and challenged with E, rCyp c 1 and F, control antigen for the mouse groups (x‐axis: 1‐5). Significant differences between the sensitized groups are indicated. (*) *P*‐value < 0.05, (**) *P*‐value < 0.01, (***) *P*‐value < 0.001

**Figure 4:**
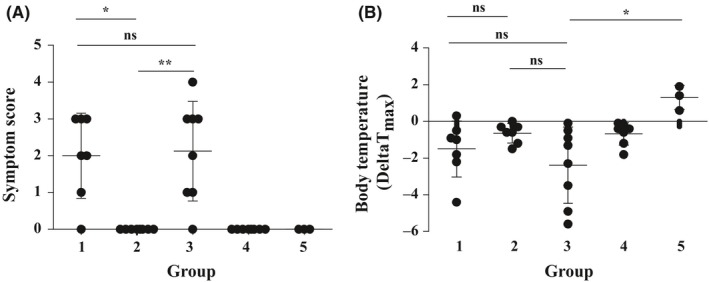
Prophylactic feeding suppresses allergic symptoms induced by challenge with nCyp c 1. Mouse groups 1‐5 received 10 mg of nCyp c 1 intragastric and A, allergic symptoms (y‐axis: mean symptom scores ± SD) and B, base line body temperatures and minimal temperatures after challenge were recorded for each mouse group (x‐axes). Maximal delta body temperatures (minimum compared to baseline T value) are shown on the y‐axis. Means ± SDs are indicated. (*P < 0.05, **P < 0.01)

**Figure 5:**
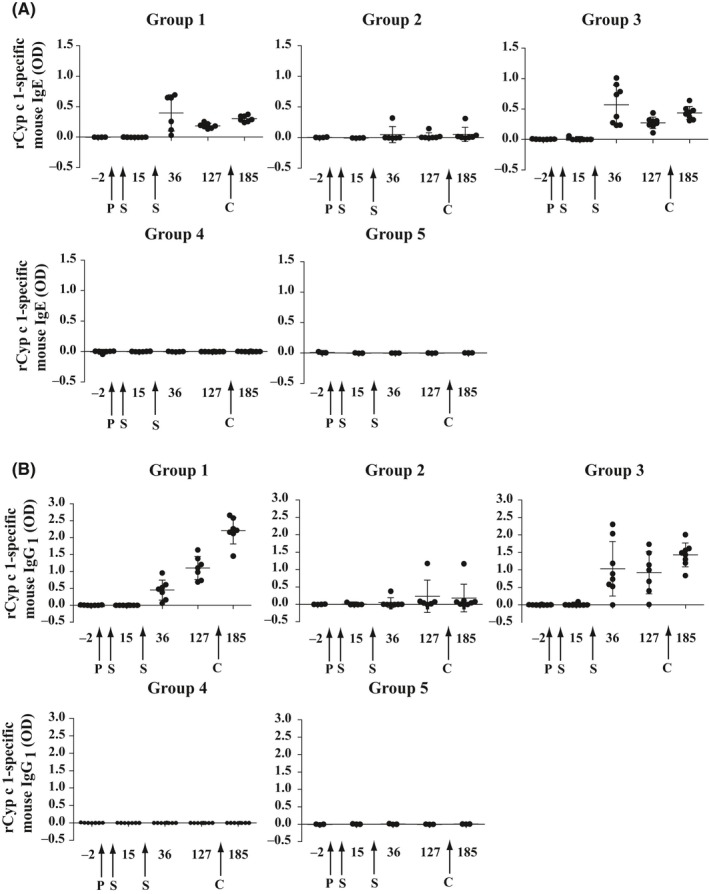
Time courses of rCyp c 1‐specific IgE and IgG_1_ antibody responses in mouse groups 1‐5. A, IgE and B, IgG_1_ antibody levels (OD, y‐axes: mean ± SD) of the individual mouse groups 1‐5 were measured in sera obtained at different time points (days, x‐axes). Interventions are indicated: *P*, prophylactic feeding; *S*, sensitization; *C*, challenge

**Figure 6:**
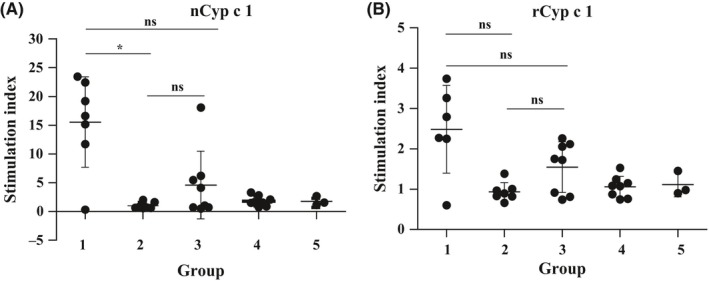
Absence of allergen‐specific T‐cell proliferation in mice prophylactically fed with nCyp c 1. Splenocyte proliferations in response to stimulation with A, nCyp c 1 and B, rCyp c 1 are shown as stimulation indices (y‐axes: SIs, means ± SDs) for mouse groups 1‐5 (x‐axes) at day 204. (*) *P*‐value < 0.05

### 3.2 nCyp c 1 shows higher resistance to pancreatic digestion as compared to mCyp c 1

The resistance of nCyp c 1 and mCyp c 1 to digestion was studied by *in vitro* gastric and pancreatic digestion assays. The proteins were incubated with a cocktail of gastric or pancreatic enzymes and samples taken at different time points were analyzed by SDS‐PAGE. nCyp c 1 (Figure 1E‐F; upper panel) and mCyp c 1 (Figure 1E‐F; lower panel) showed a similar degradation profile in the *in vitro* gastric digestion assay. By contrast, nCyp c 1 demonstrated an increased resistance against pancreatic digestion (ie, up to 45 minutes of incubation) (Figure 1F; upper panel) compared to mCyp c 1 which was completely degraded after 10 minutes (Figure 1F; lower panel). The amino acid sequences of Cyp c 1 and mCyp c 1 were analyzed regarding cleavage sites recognized by pepsin, trypsin, chymotrypsin, and carboxypeptidase A and B (Figure 1A). The point mutations in mCyp c 1 had no apparent effect on the cleavage sites (Figure 1A).

### 3.3 Allergen‐specific antibody and effector cell responses are prevented by prophylactic feeding with nCyp c 1 but not with mCyp c 1

Next, we compared the ability of nCyp c 1 and mCyp c 1 to prevent allergic sensitization to nCyp c 1 by prophylactic feeding in a mouse model of fish allergy (Figure 2A). According to the protocol given in Figure 2A, a single high‐dose feeding of either nCyp c 1, mCyp c 1, or PBS was followed by sensitization to nCyp c 1 and an unrelated control antigen or sham treatment (Figure 2B). On day 36, rCyp c 1‐ and control antigen‐specific IgE and IgG_1_ antibody responses were measured by ELISA in sera from the different mouse groups (Figure 3A‐D). BALB/c mice, which received only PBS i.g. on day 1 developed a robust Cyp c 1‐ and control antigen‐specific IgE and IgG_1_ response (group 1; Figure 3A‐D). In contrast, the Cyp c 1‐specific but not control antigen‐specific IgE and IgG_1_ antibody responses were significantly suppressed in mice fed with nCyp c 1 (group 2; Figure 3A‐D). Interestingly, mice having received mCyp c 1 i.g. before sensitization were not protected from the development of Cyp c 1‐specific antibodies (group 3; Figure 3A,B). Feeding of nCyp c 1 or mCyp c 1 alone did not induce an allergen‐specific antibody response because mice which were fed but not sensitized did not mount Cyp c 1‐specific IgE or IgG_1_ responses (groups 4 and 5; Figure 3A,B).

Next, we studied the effect of prophylactic feeding of nCyp c 1 and mCyp c 1 on IgE‐mediated immediate allergic reactions in basophil activation assays on day 80. RBL‐2H3 cells loaded with IgE from group 1 which had received only PBS i.g. and challenged with rCyp c 1 showed a mean ß‐hexosaminidase release of 33%, while there was almost no specific mediator release in group 2 which had been tolerized with nCyp c 1 (mean release: 10%) (Figure 3E). Loading of sera from mice having received mCyp c 1 before sensitization on RBL cells resulted in a mean mediator release of 45% (group 3; Figure 3E) showing that the i.g. application of mCyp c 1 did not suppress allergic sensitization. The analysis of basophil release induced with the control antigen showed that there was no significant difference between mice having received only PBS i.g. or nCyp c 1 demonstrating that the suppression of effector cell activation was indeed allergen‐specific (Figure 3F).

### 3.4 Oral tolerance induction with nCyp c 1 but not with mCyp c 1 protects against anaphylactic symptoms upon allergen challenge

In order to investigate the effects of sensitization and/or tolerance induction on symptoms of food allergy, all mouse groups were challenged by intragastric gavage with nCyp c 1 on day 177. Upon challenge, allergic symptoms and drops of body temperature indicative of systemic allergic reactions were recorded. Mice from group 1 which were sensitized to nCyp c 1 developed upon challenge allergic symptoms (mean symptom score: 2) whereas mice from group 2 which had been tolerized with nCyp c 1 did not develop any symptoms upon allergen challenge (Figure 4A). Mice from group 3 having received i.g. gavage with mCyp c 1 followed by sensitization with nCyp c 1 showed anaphylactic symptoms upon allergen challenge which were comparable to group 1 (mean symptom score: 2) (Figure 4A). Mice of groups 4 and 5 which had received only prophylactic feeding of nCyp c 1 or mCyp c 1 developed no symptoms indicating that high‐dose early feeding does not induce food allergic symptoms (Figure 4A). The severity of symptoms corresponded with the drops in body temperature measured in the mouse groups (Figure 4B). Mice from group 2 which had been tolerized with nCyp c 1 as well as mice from groups 4 and 5 which had not been sensitized showed no relevant drops of body temperature. By contrast, mice which were not tolerized (group 1) or had received mCyp c 1 (group 3) showed drops in body temperature upon intragastric allergen challenge (Figure 4B).

### 3.5 Oral tolerance induction with nCyp c 1 but not with mCyp c 1 induces long‐lasting prevention of allergen‐specific T‐cell and antibody responses

Figure 5 shows the time course of Cyp c 1‐specific IgE (Figure 5A) and IgG_1_ antibody levels (Figure 5B) for each of the mouse groups until day 185 after the allergen challenge. Cyp c 1‐specific IgE and IgG_1_ antibodies were elevated in sera from mice of group 1 which had not been tolerized and in mice from group 3 which had been tolerized with mCyp c 1 until day 185 whereas mice which had been tolerized with nCyp c 1 (group 2) and mice which had never been sensitized (groups 4 and 5) lacked relevant allergen‐specific IgE and IgG_1_ responses. A comparison of allergen‐specific IgE, IgG_1_, IgG_2a_, IgG_3_, IgM, and IgA antibody responses (Figure S2A‐F) performed on day 127 showed a significant suppression of responses in all classes and subclasses in mice from group 2 which had been tolerized with nCyp c 1 as compared to mice from group 1 which had not been tolerized and mice from group 3 which had received mCyp c 1 for tolerance induction (Figure S2).

The Cyp c 1‐specific T‐cell response was investigated on day 204. We found that no relevant nCyp c 1‐ and rCyp c 1‐specific T‐cell proliferation was observed in mice of group 2 which had been tolerized with nCyp c 1 when comparing with mice which had never been sensitized (ie, group 4 and 5) (Figure 6A,B). The nCyp c 1‐specific T‐cell proliferation in mice of group 1 was significantly higher than that in mice of group 2 (Figure 6A). Only partial suppression of splenocyte proliferation in mice of group 3 was observed (Figure 6A,B).

## 4 DISCUSSION

Our study is the first to demonstrate that the intrinsic sensitivity of an allergen to gastrointestinal digestion affects the ability of the antigen to induce robust and long‐lasting immunological and “clinical” oral tolerance. Using two forms of the major fish allergen, the digestion‐resistant wild‐type Cyp c 1 and a digestion‐sensitive mutant, mCyp c 1, for oral tolerance induction in a murine model of fish allergy, we found that only the wild‐type allergen but not the mutant form induced robust and long‐lasting immunological tolerance. In our murine study, a single high‐dose regimen was used which, of course, may need to be adapted to the human situation.

Tolerance was demonstrated by lack of allergen‐specific antibody and cellular responses as well as of “clinical” tolerance as shown by lack of anaphylactic symptoms. The specificity of oral tolerance was shown by the fact that tolerized mice lacking Cyp c 1‐specific adaptive immune responses mounted specific IgE and IgG responses against an unrelated control antigen which was used for sensitization at the same time as Cyp c 1. Therefore, bystander suppression which might be mediated by cytokines like IL‐10 and TGF‐ß secreted from regulatory T cells does not seem to play a major role. Several arguments support the assumption that the different sensitivity to digestion of wild‐type Cyp c 1 and mCyp c 1 is responsible for their different ability to induce oral tolerance. First, the mapping of the major T‐cell epitope of the two proteins with synthetic overlapping peptides suggests that the four point mutations did not affect T‐cell recognition in the mouse model. We thus assumed that the difference regarding the four amino acids did not affect the T‐cell‐based tolerogenic properties of the two antigens. Furthermore, both wild‐type Cyp c 1 and mCyp c 1 showed a similar structural fold and were recognized by antibodies raised against mCyp c 1 which also indicates that the different tolerogenic properties of the proteins cannot be due to different immunological characteristics. A tolerogenic effect of endotoxins could also be excluded, as E coli‐expressed mCyp c 1 contained higher LPS levels than natural Cyp c 1. By contrast, we found that mCyp c 1 was less resistant to enzymatic digestion. This higher sensitivity to digestion does not seem to be due to the presence of additional protease cleavage sites caused by the mutations because the analysis of the sequences of wild‐type and mCyp c 1 showed that both proteins had identical cleavage sites. We therefore think that the exchange of 4 amino acids destabilized the protein because they affect the protein's ability to bind calcium which is important for the overall stability of the protein and is known to affect the surface exposure of certain amino acids in the calcium‐bound and calcium‐free apoform of calcium‐binding proteins.^32^, ^33^ Another observation which supports our assumption that the sensitivity to digestion has affected the ability of the proteins to induce oral tolerance is the earlier finding that protection of allergens against digestion by enteric coating allowed to reduce the dose required for tolerance induction.^34^, ^35^

Our finding that sensitivity to digestion affects oral tolerance induction is important for at least two reasons. First, it may explain why not all clinical studies which have investigated oral tolerance induction had identical outcomes.^11^ In fact, different food allergen sources contain allergens with different stability and the subjects enrolled in these studies are sensitized against different allergens which may have different stability. Second and importantly, our study identifies sensitivity to digestion as another important factor for successful oral tolerance induction besides dose and timing of administration. Therefore, the development of strategies for protecting allergens or allergen‐derived molecules from proteolytic cleavage may facilitate the induction of oral tolerance.^11^

## CONFLICTS OF INTEREST

VR received grants from Biomay AG, Vienna, Austria, and Viravaxx, Vienna, Austria, and personal fees from Biomay AG, Vienna, Austria, and Viravaxx, Vienna, Austria. RvR received personal fees from HAL Allergy BV, Citeq BV, and Thermo Fisher Scientific. FS is employed by Biomay AG, Vienna, Austria. BL received personal fees from Thermo Fisher Scientific. RF received financial support from the German Society for Allergology and Clinical Immunology (DGAKI). MFT, AG, UB, IS, and TW have no conflicts of interest to disclose.

## AUTHOR CONTRIBUTIONS

BL and RV designed research; RF, AG, UB, and BL performed research and analyzed data; BL, RV, IS, and MFT supervised experiments; FS provided critical reagents; RF, RV, BL, R.v.R., and TW wrote and edited the manuscript.

## References

[1] Yu W , Freeland D , Nadeau KC . Food allergy: immune mechanisms, diagnosis and immunotherapy. Nat Rev Immunol. 2016;16:751‐765. 27795547 10.1038/nri.2016.111 PMC5123910

[2] Renz H , Allen KJ , Sicherer SH , et al. Food allergy. Nat Rev Dis Primers. 2018;4:17098. 29300005 10.1038/nrdp.2017.98

[3] Valenta R , Hochwallner H , Linhart B , Pahr S . Food allergies: the basics. Gastroenterology. 2015;148:1120‐1131. 25680669 10.1053/j.gastro.2015.02.006 PMC4414527

[4] Freeland D , Manohar M , Andorf S , Hobson BD , Zhang W , Nadeau KC . Oral immunotherapy for food allergy. Semin Immunol. 2017;30:36‐44. 28865877 10.1016/j.smim.2017.08.008 PMC5776738

[5] Yanagida N , Sato S , Ebisawa M . Clinical aspects of oral immunotherapy for the treatment of allergies. Semin Immunol. 2017;30:45‐51. 28780220 10.1016/j.smim.2017.07.008

[6] Du Toit G , Sampson HA , Plaut M , Burks AW , Akdis CA , Lack G . Food allergy: update on prevention and tolerance. J Allergy Clin Immunol. 2018;141:30‐40. 29191680 10.1016/j.jaci.2017.11.010 PMC12548800

[7] Hamad A , Burks W . Oral tolerance and allergy. Semin Immunol. 2017;30:28‐35. 28739336 10.1016/j.smim.2017.07.001

[8] Valenta R , Campana R , Marth K , van Hage M . Allergen‐specific immunotherapy: from therapeutic vaccines to prophylactic approaches. J Intern Med. 2012;272:144‐157. 22640224 10.1111/j.1365-2796.2012.02556.x PMC4573524

[9] Matricardi PM . Allergen‐specific immunoprophylaxis: toward secondary prevention of allergic rhinitis? Pediatr Allergy Immunol. 2014;25:15‐18. 24588481 10.1111/pai.12200

[10] Szépfalusi Z , Bannert C , Ronceray L , et al. Preventive sublingual immunotherapy in preschool children: first evidence for safety and pro‐tolerogenic effects. Pediatr Allergy Immunol. 2014;25:788‐795. 25406682 10.1111/pai.12310 PMC6597351

[11] Campana R , Huang H‐J , Freidl R , et al. Recombinant allergen and peptide‐based approaches for allergy prevention by oral tolerance. Semin Immunol. 2017;30:67‐80. 28939389 10.1016/j.smim.2017.08.017

[12] Ruethers T , Taki AC , Johnston EB , et al. Seafood allergy: a comprehensive review of fish and shellfish allergens. Mol Immunol. 2018;100:28‐57. 29858102 10.1016/j.molimm.2018.04.008

[13] Swoboda I , Bugajska‐Schretter A , Verdino P , et al. Recombinant carp parvalbumin, the major cross‐reactive fish allergen: a tool for diagnosis and therapy of fish allergy. J Immunol. 2002;168:4576‐4584. 11971005 10.4049/jimmunol.168.9.4576

[14] Swoboda I , Bugajska‐Schretter A , Linhart B , et al. A recombinant hypoallergenic parvalbumin mutant for immunotherapy of IgE‐mediated fish allergy. J Immunol. 2007;178:6290‐6296. 17475857 10.4049/jimmunol.178.10.6290

[15] Douladiris N , Linhart B , Swoboda I , et al. In vivo allergenic activity of a hypoallergenic mutant of the major fish allergen Cyp c 1 evaluated by means of skin testing. J Allergy Clin Immunol. 2015;136:493‐495. 25746971 10.1016/j.jaci.2015.01.015 PMC6597366

[16] Zuidmeer‐Jongejan L , Huber H , Swoboda I , et al. Development of a hypoallergenic recombinant parvalbumin for first‐in‐man subcutaneous immunotherapy of fish allergy. Int Arch Allergy Immunol. 2015;166:41‐51. 25765512 10.1159/000371657

[17] Clinical trials.gov [Internet] . Bethesda (MD): National Library of Medicine (US). 2013 Dec 23 – Identifier NCT02017626, FAST‐Fish ‐Food Allergy Specific Treatment for Fish Allergy; 2015 Jun 19 [cited 2018 Aug 21]; available from: https://clinicaltrials.gov/ct2/show/NCT02017626

[18] Clinical trials.gov [Internet] . Bethesda (MD): National Library of Medicine (US). 2015 Mar 9 ‐ Identifier: NCT02382718, FAST Fish Phase IIb Clinical Trial for the Treatment of Fish Allergy by Subcutaneous Immunotherapy (FASTIIb); 2017 Jun 12 [cited 2018 Aug 21]; available from: https://clinicaltrials.gov/ct2/show/NCT02382718

[19] Freidl R , Gstoettner A , Baranyi U , et al. Blocking antibodies induced by immunization with a hypoallergenic parvalbumin mutant reduce allergic symptoms in a mouse model of fish allergy. J Allergy Clin Immunol. 2017;139:1897‐1905. 27876628 10.1016/j.jaci.2016.10.018 PMC5438872

[20] Orengo JM , Radin AR , Kamat V , et al. Treating cat allergy with monoclonal IgG antibodies that bind allergen and prevent IgE engagement. Nat Commun. 2018;9:1421. 29650949 10.1038/s41467-018-03636-8 PMC5897525

[21] Flicker S , Linhart B , Wild C , Wiedermann U , Valenta R . Passive immunization with allergen‐specific IgG antibodies for treatment and prevention of allergy. Immunobiology. 2013;218:884‐891. 23182706 10.1016/j.imbio.2012.10.008 PMC3636530

[22] Bugajska‐Schretter A , Grote M , Vangelista L , et al. Purification, biochemical, and immunological characterisation of a major food allergen: different immunoglobulin E recognition of the apo‐ and calcium‐bound forms of carp parvalbumin. Gut. 2000;46:661‐669. 10764710 10.1136/gut.46.5.661 PMC1727915

[23] Srinivasan B , Focke‐Tejkl M , Swoboda I , et al. A combined biochemical, biophysical and immunological approach towards the identification of celiac disease‐specific wheat antigens. Amino Acids. 2013;45:889‐900. 23836418 10.1007/s00726-013-1537-6

[24] Linhart B , Mothes‐Luksch N , Vrtala S , Kneidinger M , Valent P , Valenta R . A hypoallergenic hybrid molecule with increased immunogenicity consisting of derivatives of the major grass pollen allergens, Phl p 2 and Phl p 6. Biol Chem. 2008;389:925‐933. 18627317 10.1515/BC.2008.105

[25] Handbook of Proteolytic Enzymes (Third Edition). Pshezhetsky AV: Lysosomal Carboxypeptidase A. 2013.

[26] Handbook of Proteolytic Enzymes (Third Edition). Avilés FX and Vendrell J. Carboxypeptidase B. 2013.

[27] Vieths S , Reindl J , Müller U , Hoffmann A , Haustein D . Digestibility of peanut and hazelnut allergens investigated by a simple in vitro procedure. Eur Food Res Technol. 1999;209:379‐388.

[28] Pahr S , Selb R , Weber M , et al. Biochemical, Biophysical and IgE‐Epitope Characterization of the Wheat Food Allergen, Tri a 37. PLoS One. 2014;9(11):e111483. 25368998 10.1371/journal.pone.0111483 PMC4219751

[29] Baar A , Pahr S , Constantin C , et al. Molecular and immunological characterization of Tri a 36, a low molecular weight glutenin, as a novel major wheat food allergen. J Immunol. 2012;189:3018‐3025. 22904302 10.4049/jimmunol.1200438

[30] Linhart B , Bigenzahn S , Hartl A , et al. Costimulation blockade inhibits allergic sensitization but does not affect established allergy in a murine model of grass pollen allergy. J Immunol. 2007;178:3924‐3931. 17339493 10.4049/jimmunol.178.6.3924 PMC2993922

[31] Li X‐M , Serebrisky D , Lee S‐Y , et al. A murine model of peanut anaphylaxis: T‐ and B‐cell responses to a major peanut allergen mimic human responses. J Allergy Clin Immunol. 2000;106:150‐158. 10887318 10.1067/mai.2000.107395

[32] Hayek B , Vangelista L , Pastore A , et al. Molecular and immunologic characterization of a highly cross‐reactive two EF‐hand calcium‐binding alder pollen allergen, Aln g 4: structural basis for calcium‐modulated IgE recognition. J Immunol. 1998;161:7031‐7039. 9862740

[33] Niederberger V , Hayek B , Vrtala S , et al. Calcium‐dependent immunoglobulin E recognition of the apo‐ and calcium‐bound form of a cross‐reactive two EF‐hand timothy grass pollen allergen, Phl p 7. FASEB J. 1999;13:843‐856. 10224228 10.1096/fasebj.13.8.843

[34] Fritsché R , Pahud JJ , Pecquet S , Pfeifer A . Induction of systemic immunologic tolerance to beta‐lactoglobulin by oral administration of a whey protein hydrolysate. J Allergy Clin Immunol. 1997;100:266‐273. 9275151 10.1016/s0091-6749(97)70235-5

[35] Pecquet S , Leo E , Fritsché R , Pfeifer A , Couvreur P , Fattal E . Oral tolerance elicited in mice by beta‐lactoglobulin entrapped in biodegradable microspheres. Vaccine. 2000;18:1196‐1202. 10649620 10.1016/s0264-410x(99)00384-9

