# Supplementary material for: Resistance of parvalbumin to gastrointestinal digestion is required for profound and long‐lasting prophylactic oral tolerance
Source: Allergy. 2019 Oct 3;75(2):326–35. doi: 10.1111/all.13994 (PMC7065025; doi:10.1111/all.13994)
Supplement: Supplementary file 3 [file ALL-75-326-s003.docx]

**Table S1.** Amino acid sequences of Cyp c 1-derived peptides. pI: isoelectric point; MW (Da): molecular weight (Dalton).

**Table S1.**

| **Peptide** | **Position** | **Amino acid sequence** | **pI** | **MW (Da)** |
| --- | --- | --- | --- | --- |
| 1 | 1-20 | MAFAGILNDA DITAALQGCQ | 3.56 | 2023.3 |
| 2 | 16-35 | LQGCQ AADSFDYKSF FAKVG | 5.95 | 2182.4 |
| 3 | 31-50 | FAKVGLSAKT PDDIKKAFAV | 9.53 | 2106.4 |
| 4 | 46-65 | KAFAV IDQDKSGFIE EDELK | 4.30 | 2282.5 |
| 5 | 61-80 | EDELKLFLQN FSAGARALTD | 4.32 | 2238.4 |
| 6 | 76-95 | RALTD AETKAFLKAG DSDGD | 4.36 | 2081.2 |
| 7 | 90-109 | G DSDGDGKIGV DEFAALVKA | 4.04 | 1964.1 |
